# Difficult-to-Engage Patients with Severe Mental Illness in Rural Community Settings: Results of the Greek Hybrid Assertive Community Treatment Model of Mental Healthcare

**DOI:** 10.3390/jcm13092660

**Published:** 2024-05-01

**Authors:** Fotini Tsoli, Ioanna Athina Botsari, Agnes Tsianeli, Nefeli Menti, Panagiota Kontoudi, Vaios Peritogiannis

**Affiliations:** 1 Mobile Mental Health Unit of the Prefectures of Ioannina and Thesprotia, Society for the Promotion of Mental Health in Epirus, 45445 Ioannina, Greece; ftsolh@yahoo.gr (F.T.); agnes_tsianeli@yahoo.gr (A.T.); nefeli_menti@yahoo.gr (N.M.); giotakontoy8@gmail.com (P.K.); 2 Early Intervention in Psychosis Unit, University Mental Health Research Institute, 11527 Athens, Greece; ibotsari@med.uoa.gr

**Keywords:** assertive community treatment, functioning, hospitalizations, rural areas, severe mental illness, treatment engagement

## Abstract

**Background**: Modified Assertive Community Treatment (ACT) in rural settings may be effective in the care of patients with severe mental illness (SMI) that are difficult to engage in community care. The objective of the present study was to explore the impact of the care by a hybrid ACT team on SMI patients’ hospitalizations, length of hospital stay, symptomatology and functioning in a rural community treatment setting in Greece. **Methods**: The hybrid ACT team is an expansion of the services of the well-established generic Mobile Mental Health Unit in a rural area of Northwest Greece, and delivers home-based care for patients with SMI. This was a 3-year prospective, mirror image, pre-post observational study. Patients’ symptomatology, functioning and general outcome were measured with the use of the Brief Psychiatric Rating Scale (BPRS), the Global Assessment of Functioning Scale (GAF), and the Health of the Nation Outcome Scale (HοNOS). **Results**: The mean age of the 23 enrolled patients was 52.4 years and the mean age of disease onset was 23.5 years, with a mean number of hospitalizations 10.74. Over the 16-month follow-up patients’ hospitalizations, both voluntary and involuntary, had been significantly reduced by almost 80%. Length of hospital stay had been significantly reduced by 87%, whereas patients’ functioning and symptomatology had been significantly improved, by 17% and 14.5%, respectively. **Conclusions**: The model of hybrid ACT in rural areas in Greece may be effective in the treatment of difficult-to-engage patients with SMI and may improve patients’ outcomes.

## 1. Introduction

Severe mental illness (SMI) comprises a range of persistent mental disorders, such as schizophrenia and related psychoses, as well as bipolar disorder, which may disable patients; limit their functioning; [1,2] and are difficult to manage. Care of those disorders should be community-oriented, with a focus on psychosocial rehabilitation, and various outpatient care models have been developed in this respect [3,4]. However, the provision of outpatient care may often be challenging due to clients’ disengagement from services and medication non-adherence, that may have several negative consequences, such as increased relapse rates and repeated hospitalizations [5]. With regard to service attendance, the rate of missed follow-up appointments in patients with schizophrenia has been estimated at 20–33% [6,7] and may be even higher in patients with a first episode of psychosis, resulting in poor long-term illness outcomes [8]. Notably, clients with SMI may be particularly prone to care discontinuation when referred to new services or over transitioning between levels of care; for instance, from acute inpatient treatment to community-based care [9], with non-appointment rates up to 37–60% in those recently discharged from inpatient treatment [6,7]. Accordingly, it has been reported that half of SMI patients’ readmissions occur over a few months after discharge [10].

It thus appears that a minority of clients with SMI is particularly difficult to engage in care and are in need of intensive models of care, such as the Assertive Community Treatment (ACT). ACT is an intensive mental health program in which a multidisciplinary team of professionals deliver services to clients who do not readily use clinic-based services and are at high risk for psychiatric hospitalization [11]. The original ACT model as described by Stein and Test [12] and Stein and Santos [13] is one of the most-researched and evidence-based mental health treatment models in Western countries. ACT substantially reduces psychiatric hospital use and improves clients’ symptomatology, subjective quality of life and satisfaction with services, whereas successfully engages clients in care in various settings [14,15,16]. More recently, a study in Denmark found that patients allocated to ACT had significantly fewer voluntary and involuntary admissions, as well as decreased number of contacts with psychiatric emergency departments, compared with patients attended other psychiatric services [17]. Previous research has shown a positive correlation between fidelity and outcomes of the ACT [18,19]. However, modifications to the fidelity of the original ACT model and relative adjustments may be justified, in order to adapt the model to clients’ needs in different settings [20,21,22]. Accordingly, several variants of the ACT model have been introduced and tested over the last years. A modified ACT team has been implemented in Geneva, Switzerland, and it was found to have a positive effect on patients’ symptoms and quality of life [23]. Another modified ACT model embedded in an integrated care program for patients with severe psychotic disorders has been implemented in Germany, and appeared to be effective in reducing involuntary admissions over a 4-year observation period [24]. Subsequently, in the U.S.A. less than one-fifth of the facilities with ACT reported offering all core ACT services. It has been suggested that administrative and funding differences may account for the observed variability [25]. 

ACT programs were not specifically designed for rural populations, with major barriers for their implementation being the low population density and staff shortages. However, there have been efforts to adapt this model in the rural context [26]. A previous study on the effectiveness of ACT in rural Canada reported a reduction of emergency room visits along with high satisfaction amongst clients with schizophrenia [27]. Other research on rural ACT teams has shown that their effectiveness in reducing hospitalizations may be comparable to urban teams, whereas effects on psychosocial outcomes may be less clear and inconsistent [28].

In rural Greece patients with mental disorders receive care in the community by the locally-based Mobile Mental Health Units (MMHUs), which are generic community mental health teams that deliver services according to the principles of social and community psychiatry [29]. The MMHUs are integrated within the primary healthcare system and examine all referrals, with a special focus on patients with SMI [30]. There is preliminary evidence that the MMHUs may be effective in the treatment of both severe and common mental disorders and may reduce hospitalizations and disease-related disability [31,32,33]. Although previous research on SMI patients has shown that attendance rate with the MMHUs is satisfactory [34], still a substantial proportion, that is almost one-third disengage from care. Indeed, several patients with SMI may be particularly difficult to engage in outpatient care and accordingly, over the recent years the Greek state has launched a number of hybrid ACT teams to address this issue. The basic principles of this hybrid ACT model of care in rural areas have been previously described [35], yet there are currently no data on the effectiveness of the model. The objective of the present study was to explore the impact of the model on hospitalizations and length of hospital stay. Also, to assess changes in clients’ symptomatology, functioning and general outcome. 

## 2. Materials and Methods

### 2.1. The Treatment Setting

The Mobile Mental Health Unit of the prefectures of Ioannina and Thesprotia (MMHU I-T) in Epirus, Northwest Greece, has been launched in 2007 and delivers services in a rural, mostly mountainous area of approximately 5000 km^2^ for a population grossly estimated at 100,000 inhabitants [36]. The MMHU I-T prioritizes patients with SMI and it has been shown that it can successfully engage those patients with care [34], and reduces hospitalizations and length of hospital stay [31]. The impact of the MMHU I-T on patients’ symptomatology and functioning may be less clear [37].

The hybrid ACT team was launched in December 2019, as an expansion of the services of the well-established generic MMHU I-T and delivers care for clients with SMI with a poor history of service attendance and medication adherence, as well as repeated hospitalizations in the same catchment area. It is a specialized, yet low-cost service, comprised by 4 mental health professionals (consultant psychiatrist, psychologist, social worker and nurse). The main characteristics of the hybrid ACT team are home-based interventions, a low, 1:10–12 caseload, 5 working days per week, assertive outreach and focus on engagement, and systematic contact with relatives and other caregivers [35]. Fidelity to the original ACT model is moderate, as there is no potential for extended hour operation. Similar modified mobile ACT model has been previously implemented in the city of Geneva, Switzerland [23]. The rationale for the introduction of a hybrid ACT model in the present rural catchment area derives from the notion that in places with fully staffed and well-organized community mental health services, such as the MMHU I-T, which adopt several of the core ingredients of ACT, a modified version of the latter may be reserved for patients that do not engage in care with the existing services and have multiple relapses and hospitalizations [38]. Additionally, a hybrid ACT service would be less expensive than the original model, which is relevant in low-resourced rural settings.

The hybrid ACT team accepts referrals exclusively from the psychiatric wards of the two hospitals of the area and from the MMHU I-T. Care is focused on frequent contacts and flexibility. The multidisciplinary team visits all clients on a weekly basis, and in cases of symptom exacerbation twice, or even three times a week in rare occasions. Care is delivered with the use of an intensive case management approach, that includes pharmacotherapy and psychosocial interventions, such as psychoeducation and support for the patients and their families/caregivers. Other components of care include lifestyle management, relapse prevention, rehabilitation, enhancement of selfcare and independent living, social work and employment, if possible. Medical reviews and psychosocial interventions may be performed simultaneously, or in separate contacts, according to staff’s availability. Along with the frequent face-to-face contacts, there may be in-between phone contacts, to address emerging treatment issues.

### 2.2. Study Procedures

This was a prospective, observational study. All patients with SMI that were referred to the hybrid ACT team over a 3-year period (2020–2022) were included. For the estimation of the impact of the delivered care on patients’ outcomes we applied a mirror image, pre-post comparison of patients’ outcome measures, i.e., hospitalizations, psychopathology and functioning, for the same interval prior and after engagement with the hybrid ACT care. This methodology has been previously used in research in community mental healthcare settings [24,31,32].

Data were gathered for all patients from the beginning of the program. Data-gathering took place during the first appointments with patients and involved clinical assessment and demographic information. All patients’ diagnoses were recorded by the team’s consultant psychiatrist according to the International Classification of Disease-10th revision (ICD-10). Patients’ symptomatology, functioning and general outcome were measured with the use of the Brief Psychiatric Rating Scale (BPRS) [39], the Global Assessment of Functioning Scale (GAF) [40], and the Health of the Nation Outcome Scale (HοNOS) [41], respectively. Clinician-rated scales are routinely used by the hybrid ACT team for the assessment of clinical and functional state of patients, according to the quality-of-care requirements of the service. Other clinical (disease duration, previous hospitalizations, history of alcohol/substance abuse) and demographic information were retrieved from patients’ charts.

### 2.3. Ethical Considerations

All study procedures were approved by the institutional board. Participants were all patients that attended the hybrid ACT team and no specific recruitment procedure was applied. Routinely collected data were processed anonymously for research purposes, in accordance with the general data protection regulation, that has been adopted by the Greek state. The patients’ informed consent was waived, complying with the national regulations [42,43], because no additional interventions were applied, besides the regular clinical assessment and care of patients.

### 2.4. Statistical Analysis

All statistical analyses were performed with the use of the statistical software package IBM SPSS version 27.0. Descriptive statistics were presented for the overall population and all continuous variables were presented with mean and standard deviation (SD). 

Data were checked for missing values and the assumption of normality using the Kolmogorov-Smirnov and the Shapiro-Wilk test of normality. Since the dependent variables ‘number of hospitalizations’ and ‘length of hospitalizations’ before and after the admission to hybrid-ACT model services did not meet the assumptions of normal distribution (population normality and variance equality), non-parametric tests were used to compare means and correlations between variables. For repeated measurements within the same sample (prior and after) or paired samples, the Wilcoxon Signed Rank Test was applied. A One-Sample Wilcoxon Signed Rank Test was conducted to assess whether the median of a single sample significantly differed from a specified value. A *p*-value of <0.05 was considered statistically significant.

Regarding the variables of functioning and psychopathology, specifically the GAF, HoNOS and BPRS scores over the patients’ first and last assessment by the hybrid ACT team, parametric comparison methods of means were applied, as they follow a normal distribution. These methods include t-test for dependent samples and one-way ANOVA.

## 3. Results

### 3.1. Socio-Demographic and Clinical Characteristics of the Patients

From a total of 28 referred patients over the 3-year period, sociodemographic and clinical data were retrieved for 23 service-engaged patients (13 males and 10 females). One patient did not engage in care, whereas four patients were not included in the research due to various reasons. One patient was discharged and referred to the MMHU I-T; one patient deceased; one patient was transferred to residential care; and one patient moved out of the catchment area.

The demographic and clinical characteristics of patients are presented in Table 1. Most patients were male, whereas their mean age was 52.4 years, with the age range varying between 28 and 70 years old. Patients had severe and persistent illness, and the mean age at disease onset was 23.5 years. Among the 23 patients included in the study, almost 87% had received up to 9 years of education. Regarding employment status, the majority of patients received disability benefits. In terms of family status, 60.9% were unmarried and were living with their family of origin. Patients’ diagnoses were schizophrenia (n = 10, 43.5%), schizoaffective disorder (n = 5, 21.7%) and bipolar disorder (n = 8, 34.8%). A total of 17.3% of patients had a history of alcohol/substance abuse. 

The mean follow-up of patients in the hybrid ACT team had been almost 16 months, although there were significant differences among patients (SD = 10.24). This indicates that the starting point and duration of patient care from the hybrid ACT varies widely, contributing to the open cohort nature of the service.

With regard to the patients’ medication, a large proportion was receiving antipsychotic monotherapy, whereas the antipsychotic/mood stabilizer combination was also common, according to the diagnoses and the complexity of cases. More than half of the patients were receiving benzodiazepines, but antidepressant treatment was rare (only two patients), as well as clozapine therapy (Table 2).

Patients referred to the hybrid ACT team had a history of frequent relapses and multiple hospitalizations prior to treatment engagement. Over their illness span, that had been almost 29 years, the mean number of patients’ hospitalizations was 10.74 (SD = 8.91) with a range of 1–32 hospitalizations. The mean length of hospital stay was 198.65 days (SD = 128.4), with a range of hospitalization days between 14 and 460. Compared to patients with SMI who attended the well-established MMHU I-T, patients who attended the hybrid ACT exhibited a significantly higher average hospitalization rate (10.74 vs. 2.56, respectively; z = 3.744, *p* < 0.001). This discrepancy highlights the difficulties associated with care engagement in the present sample of patients.

### 3.2. Differences in Patients’ Hospitalizations before and after Engagement in the Hybrid ACT Model

Comparisons of the variables of hospitalizations and length of hospital stay involved the same interval prior and after engagement with care for each patient. Statistical analysis showed that after engagement in care with the hybrid ACT team the number of hospitalizations had been significantly reduced (z = −3.451, *p* < 0.001), from a total average of 2.17 hospitalizations (SD = 1.78) prior to an average number of 0.43 (SD = 0.843) hospitalization with a range of 0–3 hospitalizations after (80.1% reduction). The reduction of both involuntary (z = −3.322, *p* < 0.001) and voluntary admissions (z = −1.990, *p* < 0.05) was statistically significant. After treatment engagement with the hybrid ACT service involuntary admissions were reduced from a total average of 1.96 to 0.39 or a reduction of approximately 80% (Table 3). The same applies to the length of hospital stay (z = −3.441, *p* < 0.001) that had been significantly reduced from a total average of 52.83 days of hospitalization (SD = 65.68) with a range of 1–210 days to an average number of hospitalization days of 6.91 (SD = 14.635) with a range of 1–53 days of hospitalization, or a reduction of almost 87% (Table 3).

### 3.3. Differences in Patients’ Functioning and Outcome

The patients’ functioning had been significantly improved after engagement in the hybrid ACT according to the scores in GAF scale. The t-test for paired samples in the GAF scale reveals a statistically significant difference in measurements during service attendance [t(22) = −3.919, *p* < 0.001], with a score increase from 40.43 to 47.26. There was no statistically significant difference in HoNOS scale measurements from baseline to the study endpoint [t(22) = 0.885, *p* = 0.386] (Table 3).

### 3.4. Differences in Patients’ Psychiatric Symptoms between Initial and Last Assessment

The patients’ psychiatric symptoms had been significantly improved after treatment engagement according to the scores in BPRS. The t-test for paired samples in the BPRS scale reveals a statistically significant difference in measurements between the initial and the last assessment after engagement in the hybrid ACT team [t(21) = 2.562, *p* < 0.05], with a score decrease from 42.91 to 36.68 or a 14.5% decrease. A statistically significant difference was found between diagnosis and psychopathology as measured with the BPRS scale during the first evaluation and the subsequent measurements (F = 3.992, *p* < 0.05, η2 = 0.285). The effect size (η2) demonstrates that 28.5% of the diversity in psychopathology can be linked to the diagnosis. Bipolar patients achieved lower scores on the BPRS scale compared to patients with psychotic disorders, as opposed to those with schizoaffective disorder (Table 3).

## 4. Discussion

The aim of the present study was to assess the hybrid ACT-model effectiveness related to hospitalization, length of stay, functioning, symptomatology and outcome in rural clients with SMI that had a history of poor attendance in treatment and multiple hospitalizations. To address this goal, comparisons were made in the same interval prior and after engagement in the hybrid ACT. This study found a positive impact of the hybrid ACT on the number of hospitalizations and length of inpatient stays. Improvement in patients’ symptomatology and functioning was also observed, whereas no differences were found in general outcome of patients.

The rate of patients’ engagement was high, as 27 out of 28 patients (96.4%) were initially engaged in the hybrid ACT team. This finding is in line with previous research that has consistently demonstrated that ACT teams increased and maintained patients’ contact with care [11,15]. Indeed, it has been suggested, patients who are difficult to engage with community mental health services, may find assertive community treatment more acceptable and satisfactory than standard community care [44]. In patients included in the study (n = 23) a significant decrease in hospitalizations, both voluntary and involuntary, was observed, whereas length of hospital stay was also significantly reduced, after engagement in the hybrid ACT team. This finding is an indication of the effectiveness of such care in the rural context. It is consistent with previous research concerning the original ACT model [17] and its modified versions [24], reported that ACT was effective in reducing hospital use.

The results of the present study showed that patients’ functioning had been markedly improved over the follow-up. Despite the statistically significant improvement of functioning that was recorded, the total mean score in the GAF scale was still <60, a cut-off that marks satisfactory functioning in research [45]. It is unclear if the observed improvement made a difference in patients’ every-day life, although such improvement corresponded to the clinical observations of the caring team. The overall outcome, as measured by the HoNOS remained unchanged. This may be partly explained by the structure of the HoNOS, that comprises various aspects of outcome. The relatively short follow-up may also account for the minimal changes in HoNOS scores.

With regard to patients’ symptomatology there was a statistically significant improvement in the scores of the BPRS by 14.5%, but the clinical relevance of this finding may be unclear. In clinical settings a decrease of no less than 20–30% from the initial BPRS score has been used as a cut-off for characterizing the treatment response of symptoms. Nonetheless, the widely used response cut-off of 20% does not always capture clinically significant improvement in patients with SMI [46]. Although there seems to be an association of diagnosis with symptoms’ improvement, definite conclusions cannot be drawn due to the small number of participants. A previous study of a modified ACT in Switzerland reported a great reduction of BPRS global score in patients who had high scores in study inclusion, and suggested that patients with higher BPRS scores were more likely to reach symptomatic remission when treated with ACT [23].

Generally, the main outcome that was observed in the present study involves the reduced use of hospital care, whereas modest changes were observed in terms of clinical symptoms and disability. This may not be an unexpected finding, given that the present sample comprised patients with severe and persistent mental disorders. Moreover, the period of exposure to the intervention is different for each patient, and these variables may influence the clinical and functional outcomes rather, than the outcomes concerning hospitalizations.

It seems that this less intensive, low-cost hybrid ACT model may deliver effective treatment in SMI patients, in line with other previous and more recent evidence [23,24]. Most importantly, treatment involves rural areas that generally lack specialized services and SMI research is sparse [47]. Interestingly, a previous study showed that although ACT was significantly better than standard care in sustaining contact with patients, it did not reduce admission days, nor improve psychopathology and social functioning in patients [48]. However, this study had a different design than the present study, thus comparisons cannot be made.

With regard to patients’ medication regimen, various combinations of antipsychotics and antipsychotics plus mood stabilizers had been used, according to diagnoses and the complexity of cases. A high percentage of patients received treatment with an LAI antipsychotic and a benzodiazepine, as has been previous reported in rural community treatment settings in Greece [49]. It seems surprising that only one of those patients received clozapine, however this finding indicates lack of resources and poor monitoring of clozapine treatment in remote areas. Previous research has recorded a limited clozapine prescription in rural patients with treatment-resistant schizophrenia [50].

Over the 3-year period, the number of total referred cases was 28, and could be perceived rather small. The caseload should be considered in the context of the well-established MMHU I-T, which has been shown that may effectively engage patients with SMI in care [34]. Indeed, these referrals correspond to cases with a poor history of treatment adherence and multiple hospitalizations, that otherwise would not have been engaged in any less intensive care. Another point for consideration is local geography and travelling distance. The whole area is mountainous and the population is particularly dispersed, thus the team has to travel long distances to visit patients at their homes. This may limit availability for new referrals. It has been previously suggested that ACT may not suit well to rural settings, because the number of service users requiring intensive mental health care may be small in sparsely-populated communities [51]. A recent German study reported that patients residing in areas with a lower population density and those residing in remote areas were less likely to receive intensive mental health treatment [52]. Another point to be taken under consideration is that the hybrid ACT team delivers services only over office hours, which further limits available time. Finally, the 3-year data collection took place during the COVID-19 pandemic. It has been previously shown that the COVID-19 pandemic disrupted mental health services in 93% of countries worldwide, and had a negative impact on access to mental health services [53].

### Limitations and Potential Implications

The present study has some limitations, mainly the small number of patients and the relative short follow-up. Further analysis with regard to clinical variables, such as the history of alcohol/substance abuse and the diagnosis could not be applied, due to the small number of cases. With regard to the follow-up interval, it has been previously shown that half of SMI patients’ readmissions may occur over a few months after discharge [10]. Moreover, psychiatric patients may be prone to service discontinuation when referred to new services or transitioning between levels of care, for instance, from acute inpatient to community-based care [9]. Since most referrals to the ACT team were made immediately after a hospitalization, it is relevant to include patients even with a short follow-up in the study.

The results of the study require further exploring before any conclusions can be reached. Notably, the present study involves rural clients with SMI that are difficult to engage in care with regular community mental health services, and are heavy users of inpatient treatment. It seems that the hybrid ACT model of care may improve symptomatology and functioning in patients, and if findings are replicated in larger samples, they could be relevant for clinical practice and mental healthcare policy in rural areas. Indeed, a sufficient justification for modified ACT in rural areas in Greece would be provided by a multicenter study with adequate sample size. It would be also interesting to study the hybrid ACT model in the urban context.

It is unknown whether these favorable results could be maintained after patients’ discharge and their referral to the generic MMHU I-T. Indeed, in many ACT programs there is no specific time frame for patients’ discharge [20]. For patients that are not eligible for discharge, further research is warranted in order to elucidate whether favorable outcomes are preserved in the long-term, or even further symptomatic and functional improvement occurs.

## 5. Conclusions

The model of hybrid ACT in rural areas in Greece may be effective in the care of difficult-to-engage clients with SMI and may reduce hospitalizations and hospital stays, while improving symptomatology and functioning. The results of the present study, albeit promising, require replication in different catchment areas and in larger samples. Further research is warranted to establish the effectiveness and cost-effectiveness of the hybrid ACT model in rural areas, whereas future research should comprise urban treatment settings as well.

## Figures and Tables

**Table 1:** Patients’ demographic and clinical characteristics.

Variable	Category	n	%	Mean	Standard Deviation
Gender	Female	10	43.5		
	Male	13	56.5		
Marital Status	Single	14	60.9		
	Separated	4	17.4		
	Divorced	2	8.7		
	Married	1	4.3		
	Widowed	2	8.7		
Lives With	Alone	8	34.8		
	Family of Origin	14	60.9		
	Family	1	4.3		
Educational Level	≤6 years	7	30.4		
	6–9 years	13	56.5		
	>9 years	3	13.0		
Employment Status	Disability Benefits	19	82.6		
	Unemployment	3	13.0		
	College Student	1	4.3		
Diagnosis	F20	10	43.5		
	F25	5	21.7		
	F31	8	34.8		
History of Alcohol/Substance Abuse	-	4	17.3		
Age	-	-	-	52.4	9.47
Age of Onset of the Disease	-	-	-	23.5	7.48
Duration of Patients’ Follow-up	-	-	-	15.9	10.24

**Table 2:** Patients’ medication.

Treatment Regimen	n	%
Antipsychotic monotherapy	9	39.1
Antipsychotic combination	3	13.0
Antipsychotic/mood stabilizer combination	10	43.4
Long-acting injectable antipsychotic	9	39.1
Clozapine	1	4.3
Antidepressants	2	8.7
Benzodiazepines	12	52.2

**Table 3:** Changes in patients’ hospitalizations, length of stay, symptomatology and functioning during the same interval prior and after engagement with care.

Variable	Before Care Engagement	After Care Engagement	Difference	*p*-Value
Number of Hospitalizations	2.17 (SD = 1.78)	0.43 (SD = 0.84)	80.1% Reduction	<0.001
Involuntary Admissions	1.96 (SD = 1.74)	0.39 (SD = 0.83)	80.1% Reduction	<0.001
Voluntary Admissions	0.96 (SD = 1.96)	0.17 (SD = 0.38)	82.3% Reduction	<0.05
Length of Hospital Stay (days)	52.83 (SD = 65.68)	6.91 (SD = 14.635)	86.9% Reduction	<0.001
GAF Score	40.43 (SD = 17.3)	47.26 (SD = 19.34)	16.9% Increase	<0.001
HoNOS Score	33.78 (SD = 5.4)	32 (SD = 9.75)	5.3% Decrease	0.386
BPRS Score	42.91 (SD = 9.4)	36.68 (SD = 10.58)	14.5% Decrease	<0.05
F20 Diagnosis	47.58 (SD = 9.43)	41 (SD = 8.72)	13.8% Decrease	0.089
F25 Diagnosis	38.6 (SD = 9.2)	34.2 (SD = 8.52)	11.4% Decrease	0.209
F31 Diagnosis	38.75 (6.67)	32.88 (SD = 12.95)	15.1% Decrease	0.126

SD = Standard Deviation, GAF: Global Assessment of Functioning, HoNOS: Health of the Nations Outcome Scale, BPRS: Brief Psychiatric Rating Scale, F20: Schizophrenia, F25: Schizoaffective Disorder, F31: Bipolar Disorder; *p*-values indicate the level of significance for the observed changes.

## Data Availability

Data are kept in the patients’ electronic charts of the Mobile Mental Health Unit of the prefectures of Ioannina and Thesprotia and are confidential.
